# Prevalence of Self-Reported Diabetes and Its Associated Factors: A Population-Based Study in Brazil

**DOI:** 10.1155/2015/610790

**Published:** 2015-05-18

**Authors:** Fabiana A. F. Da-Mata, Tais F. Galvao, Mauricio G. Pereira, Marcus T. Silva

**Affiliations:** ^1^Faculty of Medicine, University of Brasilia, Campus Universitario, Conjunto 16, Sala 77, 70904-970 Brasilia, DF, Brazil; ^2^Getulio Vargas University Hospital, Federal University of Amazonas, Rua Apurina, 4 Praça 14, 69020-170 Manaus, AM, Brazil; ^3^Faculty of Medicine, Federal University of Amazonas, Rua Afonso Pena 1053 Centro, 69020-160 Manaus, AM, Brazil

## Abstract

*Aim*. The aim of this study was to estimate the prevalence of diabetes and its associated risk factors in adults from Brasilia, Brazil. *Methods*. The present cross-sectional population-based study consisted of interviews with individuals aged 18–65 years. Participants were selected through two-stage probability sampling by clusters and stratified by sex and age. Demographic and clinical data were collected directly with participants from February to May 2012. Self-reported diabetes prevalence was calculated at a 95% confidence interval (CI). Prevalence ratios (PR) were adjusted by Poisson regression with robust variance. *Results*. In all, 1,820 individuals were interviewed. Diabetes prevalence in the adult population of Brasilia was 10.1% (95% CI, 8.5%–11.6%). Variables associated with diabetes were an age between 35 and 49 years (PR = 1.83; 95% CI, 1.19–2.82) or 50 and 65 years (PR = 1.95; 95% CI, 1.17–3.23), hypertension (PR = 4.04; 95% CI, 2.66–6.13), respiratory disease (PR = 1.67; 95% CI, 1.11–2.50), cardiovascular disease (PR = 1.74; 95% CI, 1.15–2.63), and pain/discomfort (PR = 1.71; 95% CI, 1.21–2.41). *Conclusion*. Diabetes is a prevalent condition in adults living in Brasilia, and disease risk increases with age and comorbidities. Future health policies should focus on screening programs and prevention for the more vulnerable groups.

## 1. Introduction

Diabetes mellitus is a global health problem and an important cause of mortality and morbidity in many countries. Its prevalence in adults has been increasing worldwide over the last 30 years [1]. It is estimated that diabetes will affect 366 million individuals worldwide by 2030 [2]. The trend of increasing diabetes prevalence seems to prevail among developing countries. In Brazil, diabetes affected 11.3 million people in 2011, and this number is likely to triple by 2030 [3]. Estimates suggest that the diabetes rate in less developed countries will increase by 69% between 2010 and 2030 [4].

Diabetes imposes a burden for society such as high socioeconomic costs that have an impact on productivity as well as life and health quality [5]. This situation seems to be worse in developing countries, where the healthcare system often fails to meet demand [6]. Studies have concluded that a Western dietary pattern, sedentary lifestyle, and genetic factors play a central role in diabetes development [7].

The Brazilian Ministry of Health has followed the World Health Organization's recommendations and has taken some actions to monitor diabetes such as an annual telephone-based survey [8]. Socioeconomic disparities might contribute to some degree of heterogeneity in measures of prevalence between regions [9]. A study demonstrated that diabetes prevalence across the Brazilian states ranged from 11% to 25%, with an overall rate of 16% in 2001 [10].

Brasilia, the capital of Brazil, is located in the Central-West region of the country. The city has the highest Human Development Index in Brazil, but it has one of the highest levels of social inequality compared with other Brazilian regions [11, 12]. These characteristics of Brasilia warrant further investigation in many aspects, including the health status of its population.

Thus, the goal of this study was to estimate the prevalence of diabetes and its associated risk factors in adults of Brasilia, Brazil.

## 2. Materials and Methods

### 2.1. Study Design and Settings

The present cross-sectional population-based study was conducted in Brasilia, Brazil, from February to May 2012. The target population was 1,702,419 inhabitants aged 18–65 years [12].

### 2.2. Sample Size and Participants

The sample size was calculated based on an estimation of 16% of self-reported diabetes cases [10]. Considering a 95% confidence interval (CI), precision of 2.25%, and a design effect of 1.8, the estimated sample size was 1,835 individuals. We added 10% of the sample size to compensate for any eventual attrition, which resulted in a final sample of 2,019 individuals.

Participants were selected by a two-stage probability sampling process by cluster and were stratified by sex and age. A total of 220 census tracts were randomly selected from 3,886 urban tracts with more than 200 inhabitants [12]. Up to 10 households were selected from each census tract. In total, one adult per household was selected following the predefined quotas of sex and age to answer the interview. Trained professionals surveyed all of the participants in their homes using a semistructured questionnaire. To ensure reliability, 20% of the interviews were audited by telephone. To test the understanding and acceptability of the questionnaire, 150 pilot interviews were held prior to data collection.

### 2.3. Study Variables

The dependent variable was self-reported diabetes. Independent variables included demographic characteristics (age group, sex, marital status, living arrangements, and household location), socioeconomic characteristics (level of education, occupation, and social class), chronic health conditions (self-reported hypertension, depression, respiratory diseases, cardiovascular diseases, and other chronic diseases), access to healthcare (health insurance, medical consultation, and hospitalization), and perceived health status (mobility, self-care, usual activities, pain/discomfort, and anxiety/depression) [13]. The stratification was based on the Brazilian criterion of economic classification, which defines five classes, with “A” being the wealthiest group and “E” being the poorest [14].

### 2.4. Statistical Analysis

In all of the analyses, the effects of complex sampling were considered. First, we described participant characteristics by weighted frequencies. Self-reported diabetes prevalence in the population was then calculated at a 95% CI. To identify factors related to diabetes prevalence, we calculated prevalence ratios (PR) using bivariate analysis and calculated the adjusted PR by a Poisson regression model with robust variance [15]. In this model, all of the variables were analyzed simultaneously. We preferred to use this more conservative model that included all of the variables to allow for better confounding adjustment. Other models that included only the most significant variables were tested and did not change the significance of the variables. Associations were considered to be statistically significant when *P* < 0.05. The STATA software version 10.1 was used for all of the calculations [16].

### 2.5. Ethics Statement

This study was approved by the University of Brasilia Ethics Committee. All participants signed a term of free and informed consent.

## 3. Results

### 3.1. Participants and Sample Characteristics

**Figure 1:**
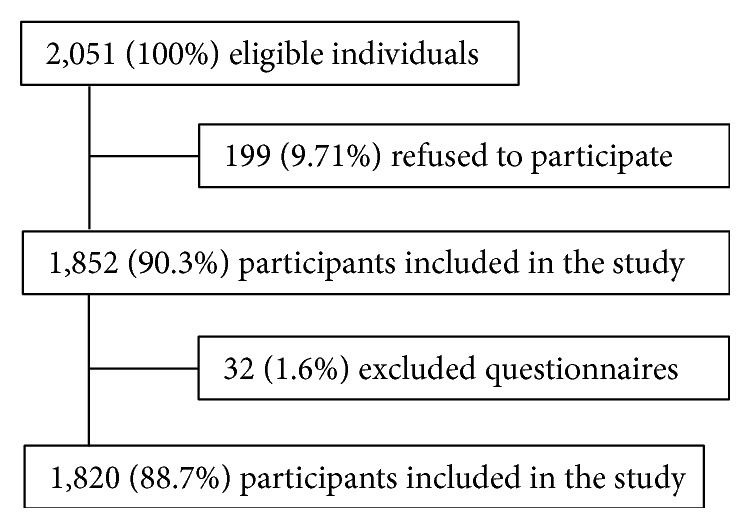
Sample selection.

In total, 1,820 individuals were included in the study (Figure 1). The main characteristics of the sample are shown in Table 1. Approximately 60% of the participants were women, and 57% were aged between 35 and 60 years. Most of the participants belonged to economic class “C,” had completed high school, were married or cohabitating, lived with at least one more person in the household, and dwelled in a satellite town.

### 3.2. Diabetes and Correlates

Diabetes was self-reported by 10.1% (95% CI: 8.5%–11.6%) of the adult population in Brasilia. Table 1 depicts diabetes prevalence and prevalence ratios (PR) before and after adjustment by Poisson regression.

The age group of 35–65 years, hypertension, respiratory disease, cardiovascular disease, and pain/discomfort were significantly associated with diabetes. Sex, marital status, living arrangements, social class, education level, employability, living location, health insurance, medical consultation, hospitalization, physical mobility, self-care, usual activities, and anxiety/depression revealed no significant association.

**Figure 2:**
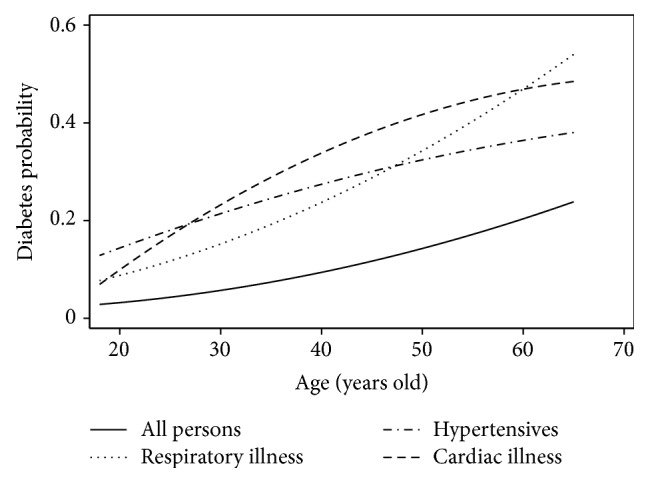
Diabetes prevalence by age in groups with different health conditions.

Figure 2 illustrates differences in diabetes prevalence between all persons and the population with comorbidities. Diabetes prevalence in the age range 30–65 years is higher among individuals with cardiovascular disease, followed by those with hypertension and those with respiratory diseases. This result suggests that the likelihood of diabetes increases with age and is greater in persons with comorbidities.

## 4. Discussion

Diabetes was self-reported by one of every ten Brazilian adults. An age of 35 years and over, presence of pain or discomfort, cardiovascular disease, hypertension, and respiratory disease were positively associated with diabetes in the adult population of Brasilia.

The main limitations of our study were the self-reported assessments of the primary outcome and independent variables. Self-reported diabetes might be a source of bias because individuals need to be aware of the diagnosis prior to answering, which could result in disease underestimation [1]. However, performing a clinical test for diagnosing diabetes is not always possible in population-based studies. Thus, self-reported answers regarding diabetes have been a common practice according to the literature [17, 18]. Another shortcoming was the cross-sectional design of the study, which hampers a causal relationship between diabetes and the significantly associated factors identified herein.

A previous population-based study developed in Brazil in 2008 used telephone interviews to investigate self-reported diabetes prevalence and found low prevalence rates in Brasilia [19]. Another study found that Brasilia was the region with the highest diabetes prevalence compared with other Brazilian regions from 2002 to 2007 [20]. Research identified a significant increase in self-reported diabetes in the Brazilian population because it ascended from 3.3% in 1998 to 5.3% in 2008 [3]. In South and Central America, the estimated diabetes prevalence in 2013 was 8.0%; Brazil demonstrated the highest prevalence, followed by Colombia and Argentina [21]. The variability of diabetes prevalence may be due to a poorer diet and a lack of physical activity, or it could be related to better access to diagnostic testing [3].

As expected, our results demonstrated that the likelihood of having diabetes increases with age. From a healthcare policy perspective, diabetes prevention and management programs should target young people and not only the elderly population.

Diabetes prevalence was higher among individuals with cardiovascular disease, hypertension, and respiratory disease compared with the general population. There is convincing evidence of the association between diabetes and hypertension, which increases the risk of a cardiovascular event [22]. A 2003 study conducted in São Luis, a city located in one of the poorest areas of Brazil, observed a positive association between diabetes and hypertension [23]. A cross-sectional study conducted between 2004 and 2005 in São Jose do Rio Preto, a city in the Brazilian southeast region, revealed that the diabetes prevalence was almost threefold higher in a population of hypertensive individuals compared with the general population [9].

A cohort study performed in women between 1988 and 1996 throughout 11 states in the United States found that chronic obstructive pulmonary disease was a diabetes risk factor [24]. A retrospective cohort study conducted in northern California reported that individuals with diabetes are at a greater risk of developing asthma, chronic obstructive pulmonary disease, fibrosis, and pneumonia [25].

Socioeconomic factors were not associated with diabetes in our sample. In contrast, a systematic review of 10 studies suggests that growing up in a socioeconomically disadvantaged environment may contribute to diabetes in later life [26]. An Australian study also described a positive association between socioeconomic variables and diabetes in adults aged 45 years and over [27].

The perceived health dimensions physical mobility, self-care, usual activities, and anxiety/depression were not associated with diabetes in our sample. In 2012, a literature review found that diabetes was considered a potential risk factor for the poor performance of daily life activities among individuals aged 50 years and over [28]. A study conducted with older adult New York residents observed that self-reported diabetes was not associated with depression [29]. Other than depression, this finding might depict an association between diabetes and activities of daily living, which may be developed at older ages.

## 5. Conclusion

Diabetes is a common health condition in adults living in Brasilia and is positively associated with older age, cardiovascular disease, hypertension, respiratory disease, and presence of pain or discomfort. Preventive strategies should prioritize populations with at least one of the identified factors.

## Figures and Tables

**Table 1:** Sociodemographics of the sample population, diabetes prevalence, and unadjusted and adjusted prevalence ratios (PR) (*N* = 1,820).

Variables	Frequency	Diabetes	Unadjusted PR	*P* value	Adjusted PR	95% CI	*P* value
	distribution (%)	prevalence (%)					
Sex							
Male	40.7	9.6	1.00	—	1.00	—	—
Female	59.3	10.4	1.08	0.641	0.89	0.65–1.23	0.489
Age group (years)							
18–34	43.5	4.5	1.00	—	1.00	—	—
35–49	35.1	11.3	2.52	<0.001	1.83	1.19–2.82	0.006
50–65	21.4	19.0	4.24	<0.001	1.95	1.17–3.23	0.010
Marital status							
Single	47.8	7.9	1.00	—	1.00	—	—
Married/cohabitating	52.2	12.0	1.52	0.014	1.61	1.16–2.72	0.005
Living arrangements							
At least with one person	94.5	10.2	1.00	—	1.00	—	—
Alone	5.5	8.1	0.79	0.529	1.02	0.49–2.12	0.954
Social class							
Class A	8.5	7.6	1.00	—	1.00	—	—
Class B	34.4	10.1	1.33	0.373	1.72	0.83–3.65	0.145
Class C	47.5	10.8	1.42	0.257	1.47	0.67–3.22	0.331
Classes D-E	9.5	8.6	1.14	0.750	1.21	0.45–3.24	0.709
Level of education							
College or higher	17.4	8.5	1.00	—	1.00	—	—
High school	34.4	8.0	0.95	0.833	0.91	0.54–1.52	0.715
Primary school	21.6	9.3	1.10	0.738	1.19	0.65–2.16	0.567
Incomplete primary school	26.6	14.5	1.72	0.034	1.07	0.58–2.00	0.826
Occupation							
Employed	45.6	8.3	1.00	—	1.00	—	—
Unemployed or retired^a^	54.4	11.6	1.40	0.055	0.97	0.70–1.35	0.877
Location							
Downtown	17.2	8.0	1.00	—	1.00	—	—
Satellite towns	82.8	10.5	1.31	0.284	0.98	0.53–1.79	0.937
Self-reported chronic conditions							
Hypertension	21.5	29.9	6.43	<0.001	4.04	2.66–6.13	<0.001
Respiratory disease	7.3	20.9	2.37	<0.001	1.67	1.11–2.50	0.013
Cardiovascular disease	6.9	36.5	4.74	<0.001	1.74	1.15–2.63	0.009
Other chronic diseases	8.0	10.6	1.05	0.828	0.54	0.29–1.01	0.052
Healthcare services							
No health insurance	72.3	9.5	0.83	0.311	0.84	0.59–1.20	0.339
Medical consultation	42.5	12.8	1.66	<0.001	0.94	0.70–1.27	0.690
Hospitalization	9.9	16.6	1.80	0.005	1.43	0.98–2.10	0.062
Perceived health status							
Mobility	7.9	19.7	2.15	<0.001	1.29	0.84–2.00	0.242
Self-care	4.0	17.7	1.83	0.045	0.75	0.35–1.63	0.471
Usual activities	6.9	16.2	1.68	0.042	0.98	0.54–1.78	0.941
Pain/discomfort	37.0	15.7	2.30	<0.001	1.71	1.21–2.41	0.002
Anxiety/depression	23.0	14.8	1.70	0.001	1.06	0.70–1.63	0.777

Note: ^a^included students not formally employed.

CI: confidence interval.

## References

[1] Rubinstein A., Gutierrez L., Beratarrechea A., Irazola V. E. (2014). Increased prevalence of diabetes in Argentina is due to easier health care access rather than to an actual increase in prevalence. *PLoS ONE*.

[2] Wild S., Roglic G., Green A., Sicree R., King H. (2004). Global prevalence of diabetes: estimates for the year 2000 and projections for 2030. *Diabetes Care*.

[3] Schmidt M. I., Duncan B. B., E Silva G. A. (2011). Chronic non-communicable diseases in Brazil: burden and current challenges. *The Lancet*.

[4] Shaw J. E., Sicree R. A., Zimmet P. Z. (2010). Global estimates of the prevalence of diabetes for 2010 and 2030. *Diabetes Research and Clinical Practice*.

[5] Bosi P. L., Carvalho A. M., Contrera D. (2009). Prevalência de diabetes melito e tolerância à glicose diminuída na população urbana de 30 a 79 anos da cidade de São Carlos, São Paulo. *Arquivos Brasileiros de Endocrinologia & Metabologia*.

[6] Almeida-Filho N. (2011). Higher education and health care in Brazil. *The Lancet*.

[7] Garcia-Dominic O., Lengerich E. J., Camacho F. (2014). Prevalence of diabetes and associated obesity in Pennsylvania adults, 1995–2010. *Preventing Chronic Disease*.

[8] Iser B. P. M., Claro R. M., de Moura E. C., Malta D. C., Neto O. L. M. (2011). Risk and protection factors for chronic non communicable diseases by telephone survey—VIGITEL Brazil—2009. *Revista Brasileira de Epidemiologia*.

[9] Cipullo J. P., Martin J. F. V., Ciorlia L. A. D. S. (2010). Hypertension prevalence and risk factors in a Brazilian urban population. *Arquivos Brasileiros de Cardiologia*.

[10] Nucci L. B., Toscano C. M., Maia A. L. M. (2004). A nationwide population screening program for diabetes in Brazil. *Revista Panamericana de Salud Publica*.

[11] Programa das Nações Unidas para o Desenvolvimento. Atlas do Desenvolvimento Humano no Brasil 2013. http://atlasbrasil.org.br/2013/ranking.

[12] IBGE- Instituto Brasileiro de Geografia e estatística. Censo Demográfico 2010. http://www.ibge.gov.br/home/estatistica/populacao/censo2010/default.shtm.

[13] Rabin R., De Charro F. (2001). EQ-5D: a measure of health status from the EuroQol Group. *Annals of Medicine*.

[14] CCEB Associação Brasileira de Empresas de Pesquisa. Critério de Classificação Econômica Brasil. 2012. http://www.abep.org/novo/Content.aspx?ContentID=139.

[15] Lee J., Chuen S. T., Kee S. C. (2009). A practical guide for multivariate analysis of dichotomous outcomes. *Annals of the Academy of Medicine Singapore*.

[16] Siller A. B., Tompkins L. (2005). The big four: analyzing complex sample survey data using SAS, SPSS, STATA, and SUDAAN.

[17] de Almeida L. A. B., Pitanga F. J. G., Freitas M. M., Pitanga C. P. S., Dantas E. H. M., Beck C. C. (2012). Caloric expenditure of different domains of physical activity as predictors of the absence of diabetes in adults. *Revista Brasileira de Medicina do Esporte*.

[18] Borges T., Rombaldi A. J., Corrêa L. Q., Knuth A. G., Hallal P. C. (2012). Prevalência de autorrelato da morbidade e conhecimento sobre diabetes: estudo populacional de uma cidade no sul do Brasil. *Revista Brasileira de Cineantropometria e Desempenho Humano*.

[19] Brasil Ministério da Saúde (2009). *Vigitel Brasil 2008: vigilância de fatores de risco e proteção para doenças crônicas por inquérito telefônico*.

[20] Dias J. C. R., Campos J. A. D. B. (2012). Diabetes mellitus: razão de prevalências nas diferentes regiões geográficas no Brasil, 2002–2007. *Ciência & Saúde Coletiva*.

[21] International Diabetes Federation (2013). *Diabetes Atlas*.

[22] Grundy S. M., Benjamin I. J., Burke G. L. (1999). Diabetes and cardiovascular disease: a statement for healthcare professionals from the american heart association. *Circulation*.

[23] Barbosa J. B., da Silva A. A. M., dos Santos A. M. (2008). Prevalência da hipertensão arterial em adultos e fatores associados em São Luís—MA. *Arquivos Brasileiros de Cardiologia*.

[24] Rana J. S., Mittleman M. A., Sheikh J. (2004). Chronic obstructive pulmonary disease, asthma, and risk of type 2 diabetes in women. *Diabetes Care*.

[25] Ehrlich S. F., Quesenberry C. P., van den Eeden S. K., Shan J., Ferrara A. (2010). Patients diagnosed with diabetes are at increased risk for asthma, chronic obstructive pulmonary disease, pulmonary fibrosis, and pneumonia but not lung cancer. *Diabetes Care*.

[26] Tamayo T., Christian H., Rathmann W. (2010). Impact of early psychosocial factors (childhood socioeconomic factors and adversities) on future risk of type 2 diabetes, metabolic disturbances and obesity: a systematic review. *BMC Public Health*.

[27] Shamshirgaran S. M., Jorm L., Bambrick H., Hennessy A. (2013). Independent roles of country of birth and socioeconomic status in the occurrence of type 2 diabetes. *BMC Public Health*.

[28] Petrofsky J., Berk L., Al-Nakhli H. (2012). The influence of autonomic dysfunction associated with aging and type 2 diabetes on daily life activities. *Experimental Diabetes Research*.

[29] Palta P., Golden S. H., Teresi J. A. (2014). Depression is not associated with diabetes control in minority elderly. *Journal of Diabetes and Its Complications*.

